# Statistical Analysis of Digital 3D Models of a Fossil Tetrapod Skull from µCT and Optical Scanning

**DOI:** 10.3390/s25196084

**Published:** 2025-10-02

**Authors:** Yaroslav Garashchenko, Ilja Kogan, Miroslaw Rucki

**Affiliations:** 1 Kharkiv Polytechnic Institute, National Technical University, 2 Kyrpychova Str., 61002 Kharkiv, Ukraine; omsroot@kpi.kharkov.ua; 2 Museum für Naturkunde Chemnitz, Moritzstr. 20, 09111 Chemnitz, Germany; kogan@naturkunde-chemnitz.de; 3 Faculty of Mechanical Engineering, Casimir Pulaski Radom University, Stasieckiego Str. 54, 26-600 Radom, Poland; 4 Institute of Mechanical Science, Vilnius Gediminas Technical University, Sauletekio al. 11, LT-10223 Vilnius, Lithuania

**Keywords:** optical surface scanning, computed microtomography, model fidelity, vertebrate paleontology, chroniosuchia

## Abstract

The quality of digital 3D models of fossils is important from the perspective of their further usage, either for scientific or didactical purposes. However, fidelity evaluation has rarely been attempted for digitized fossil objects. In the present research, a 3D triangulated model of the unique skull of *Madygenerpeton pustulatum* was built using an YXLON µCT device. The comparative analysis was performed using models obtained from seven optical surface-scanning systems. Methodology for accuracy assessment involved the determination of distances between the points in pairs of models, interchanging the reference and tested ones. Statistical significance testing using paired *t*-tests was performed. In particular, it was found that the YXLON µCT model was closest to the one obtained from AICON SmartScan, exhibiting an average distance of $\bar{{∆}_{d}}$ = −0.0183 mm with a standard deviation of σ{∆_d_} = 0.0778 mm, which is close to the permissible error of 20 µm given in technical specifications for AICON scanners. It was demonstrated that the analysis maintained measurement validity even though the YXLON model consisted of 23.8 M polygons and the AICON model consisted of 13.9 M polygons. Comparison with other digital models demonstrated that the fidelity of the triangulated µCT model made it feasible for further research and dissemination purposes.

## 1. Introduction

Nowadays, the development of technologies reaches far beyond purely engineering applications. Industrial enterprises and engineers solve technical problems faced by the general public and by researchers in fields of non-technical sciences. One example is the study of the application of scanning technologies for the geometrical analysis of objects that belong to the paleontological domain. In the 21st century, scientific treatment of fossil specimens has greatly changed due to the development of digital technologies. The external morphology of fossils, previously only accessible by verbal description, direct measurement, photography, and drawing, can now be captured at a high precision by means of photogrammetry or 3D scanning. However, application of both methods in archeology or paleontology has some advantages and disadvantages [1]. The way that fossils are studied is rapidly transforming through application of these techniques, allowing for really revolutionary computational approaches [2]. Generative models based on AI and large datasets even allow for hypothesizing and reconstruction of elements damaged or lost during fossilization processes [3]. The internal morphology of fossils, formerly approachable only by invasive techniques such as grinding or thin sectioning, can in the most cases be detected using computed microtomography (µCT) [4]. Crucially, all these methods are non-destructive and allow for obtaining morphological information on unique specimens without the risk of damaging them. Further, the digital models can be used for direct visualization in 3D educational presentations, for detailed scientific analysis, or for the reproduction with additive manufacturing techniques.

The applicability of technology depends on its availability, i.e., economic constraints. While photogrammetry is a low-cost method requiring a (not necessarily professional) digital camera, software (with freeware solutions available along with commercial ones), and a sufficiently powerful computer for post-processing, 3D scanning hard- and software equipment is more expensive by one or two orders of magnitude. The costs for computed microtomography systems are another order of magnitude higher. Being a standard method for obtaining sections and 3D models of internal structures hidden by rock or within the fossil, this methodology has seldom been used for the creation of surface models of whole specimens. For instance, Mouro et al. [5] found it useful for identification of microfossils, while Kundrata and colleagues [6] used microtomography to reveal a unique morphology in a beetle from Eocene. Costeur and co-authors [7] emphasized the importance of microtomography in providing researchers with first-hand scientific data.

However, quite a few studies have compared the effort of producing 3D surface models of fossils and the quality of the result (e.g., reported in [8,9,10,11,12]), and no more than a handful of papers have focused on contrasting fundamentally different working principles such as µCT or clinical CT and photogrammetry or surface scanning, e.g., [13,14]. Noteworthy attempts were made in testing accuracy and comparability of digital 3D models of human bones generated by CT and laser scanners [15]. Giménez-El-Amrani and co-authors [16] reported the results of a quantitative analysis of three non-contact 3D scanning technologies: structured light scanning (SLG), laser scanning (LAS), and photogrammetry (PHG). They demonstrated that these technologies generally underestimated the original size of the scanned tissues. Bilušić and Olivari [17] showed that the accuracy of optical 3D measurements is affected by a lot of various factors and parameters, which remain insufficiently researched. Thus, the available research reports provide strong motivation for fidelity analysis of freeform surfaces obtained from scanners.

In this paper, we expand our previous comparative studies on 3D surface models of a fossil vertebrate skull obtained by various techniques (photogrammetry, structured-light 3D scanning, laser scanning, and coordinate measuring machines reported in [9,12]) to include µCT scanning.

The object of our study is the holotype of the chroniosuchian *Madygenerpeton pustulatum* described by Schoch et al. [18]. This somewhat deformed skull, lacking the lower jaw, belongs to a reptiliomorph amphibian from the mid-Triassic fossil lagerstatte of Madygen (Kyrgyzstan, Central Asia). Besides the skull, the taxon is represented by parts of the dorsal carapace referrable to at least three individuals. The semi-aquatic *Madygenerpeton* was a 60–75 cm long apex predator in the lacustrine ecosystem of Madygen [19], one of the world‘s richest non-marine finding localities for Triassic vertebrates, invertebrates, and plants. The Madygen lagerstatte is the main attraction within the Madygen Geopark, one of the first perspective UNESCO Global Geoparks to be established in Central Asia. Thus, 3D visualization and replication of *Madygenerpeton* and other fossil organisms of the assemblage is not only of scientific value, but also of interest for communicating the scientific findings to students, tourists, and local authorities.

The aim of the present work is to broaden and re-evaluate previous fidelity assessments of digital 3D models by means of comparative statistical analysis. The analysis helps establish reliable algorithms for research on specific geometrical features of fossils. Since there is no available source model of the complex surface hidden inside the object, comparison was performed using the digital models obtained from the optical scanners and analyzed elsewhere [12]. In the present study, key novel contributions can be listed, as follows:

- Methodological breakthrough, i.e., demonstration that µCT can simultaneously capture internal morphology and achieve surface fidelity comparable to dedicated optical scanners;
- Quantitative validation and statistical tests proved that µCT model surface accuracy, with a value of $\bar{{∆}_{d}}$ = −0.0183 mm, approached the permissible error given in industrial optical scanner specifications, e.g., 20 µm for AICON scanners;
- Unique capability demonstrated through digital fossil–sediment separation, impossible with any optical method.

## 2. Materials and Methods

Unlike most industrial measurement tasks, where the correct or ideal geometry is known, fidelity analysis of a freeform surface can be based on several digital models, scanned by a device with some uncertainty. Having the models obtained from many scanning devices, it should be decided which one is more accurate and reflects the geometry with higher fidelity. There is no metrological reference standard or CAD model to be compared with the obtained digital 3D model. More specifically, the geometrical features of a paleontological object cannot be calculated or predicted as can be performed when solving many reverse engineering tasks. Metrological analysis cannot be based on tolerances, since no geometry specification for a fossil skull is available. To solve the fidelity assessment problems and to overcome these challenging limitations, the following test campaign was performed:

- From the scanned data of the *Madygenerpeton* skull obtained from the YXLON µCT device, the digital model was generated at maximum settings (with a model formation step 0.05 mm).
- Visual and statistical analyses of the distances between 3D digital models of the surfaces were performed using the available data from 7 different scanners.
- Detailed statistical analysis of the deviations was carried out in order to reveal the specific features of the µCT model obtained from scanning of a complex freeform object like a fossil skull.

The presented approach aligns with established practice for multi-system comparative validation in the absence of absolute reference standards.

**Figure 1:**
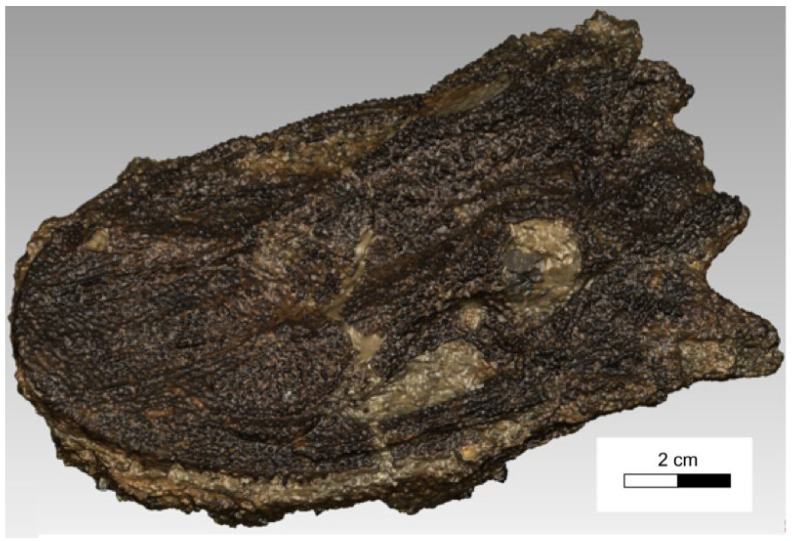
Visualization of the scanned *Madygenerpeton* skull surface.

Dimensions of the examined *Madygenerpeton* skull were ca. 10 cm × 7 cm × 3 cm. Visualization of the scanned object is shown in Figure 1. The picture was taken with a Fujifilm X-T2 camera equipped with a Fujinon Super EBC XF 10–24 mm 1:4 R OIS lens. The 3D digital model in the form of a photo-realistic textured mesh was obtained using the 3DF Zephyr commercial software package version 8.029 through processing of a point cloud via surface triangulation. This model exhibited good photographical effects but did not meet requirements for further testing.

The tomography of the *Madygenerpeton* fossil skull was made using an YXLON FF35-CT system available at the Museum für Naturkunde Berlin, Germany. The device employed a dual-tube technology, where the combination of two tubes, with a 225 kV microfocus and 190 kV nanofocus, with application of a special YXLON flat detector, provided a resolution up to 150 nm. Data acquisition parameters were as follows:

- Voxel size: 0.05 mm, isotropic;
- Projections: 1440 (0.25° steps, 360° rotation);
- Exposure: 250 ms per projection;
- Voltage/current: 160 kV, 70 µA;
- Filtering: 0.25 mm Cu beam hardening filter;
- Source-to-object distance: 89 mm; object-to-detector distance: 1200 mm;
- Reconstruction: Feldkamp–Davis–Kress (FDK) algorithm;
- Detector calibration: daily flat-field correction with 2-point gain;
- Scale calibration: ruby sphere phantom (Ø 3.000 ± 0.001 mm, traceable to PTB).

The most significant uncertainty sources include the voxel size scaling, reconstruction algorithms, segmentation threshold selection, surface triangulation parameters, and registration alignment accuracy. Each of them contributed to the final freeform model fidelity. The measured standard deviations of 0.08–0.58 mm represent the practical measurement uncertainty that can be expected for similar objects. In the research, data analysis software VGStudio MAX 3.5.2 was used with the following parameters:

- Thresholding: Otsu automatic + manual refinement (threshold = 45,000 HU);
- Morphological operations: 3 × 3 × 3 closing kernel, 2 iterations;
- Manual edits: below 2% volume, isolated voxel removal only;
- Isosurface: marching cubes algorithm;
- Smoothing: Laplacian filter, λ = 0.3, 5 iterations;
- Mesh decimation: target edge length 0.1 mm;
- Hole filling: enabled for gaps up to 0.5 mm diameter.

The respective data of the scanned object are available in the database [20]. They consist of the following files:

- Raw meshes: *.stl format, mm units, RAS coordinate system;
- Distance maps: .csv format with XYZ coordinates + distance values;
- Analysis scripts: Python 3.9 + Open3D library;
- Metadata: JSON format with acquisition parameters. License: CC BY 4.0, Version 1.2.

In order to contextualize the observed errors, triangle edge length statistical analysis was performed. The results are shown in Table 1.

The polygonal model of the µCT image was compared with the ones obtained from the optical scanners. Due to the unique character of the specimen and lack of metrological reference surface, comparison was made on the base of previous analysis and rating. The devices used were AICON, AR Strato, AR Crysta, ARTEC, CREAFORM HandyScan, CREAFORM GoScan, and EinScan Pro, described in detail in [12]. All the measurements and analyses were performed in accordance with ISO 10360-5 CMM standards [21] and ISO 17025 measurement traceability [22]. Table 2 presents data on accuracy and traceability of the devices.

Among them, the AICON model was rated as the most accurate model, close to 6 other ones. Nevertheless, in the present comparative study, all the previous models were used again. The fidelity was assessed from the comparison of distances between the model surfaces using CAD systems Geomagic Studio and Autodesk PowerShape. These programs provided the tools for polygonal models processing, as well as for the statistical and visual analysis of the deviations of the tested model from the reference one. Alignment was made automatically considering the minimum square root distances between the reference 3D model and the tested one. The two surfaces were aligned in two steps. First, the point-to-point method was applied, and then 2000 points evenly distributed on the surface were used.

The basic characterization of the polygonal models YXLON, AICON, and Mitutoyo is presented in Table 3.

It should be noted that the models obtained from Mitutoyo AR Crysta and AR Strato covered only the upper surface of the scanned object. As a result, the respective model surfaces are significantly smaller than those of other devices, and the volumes were not calculated. For these two models with partial coverage, common ROI restriction methodology was applied. To handle the issue of partial coverage, the following was applied:

- ROI definition protocol: generated 3D intersection volumes using Boolean operations in Geomagic Studio;
- Common coverage area: 68.2% of total skull surface (primarily dorsal and lateral regions);
- Excluded regions: ventral surface, posterior occipital area, broken edges;
- ROI boundaries defined by *Z*-axis threshold: Z > −15.2 mm in the skull coordinate system;
- Masking implementation: consistent 3D masks were applied to all scanner datasets before statistical analysis;
- Verified mask accuracy: below 0.1 mm boundary variation between datasets;
- Statistical validation: full-surface vs. masked comparisons showed less than 3% difference in mean distances.

It is noteworthy that the YXLON model contained almost two times more polygons than AICON did, while the volumes differed by less than 5%. The surface area larger by ca. 12% indicated more complex and detailed reproduction of the object’s surface by the larger number of the polygons.

## 3. Results and Discussion

**Figure 2:**
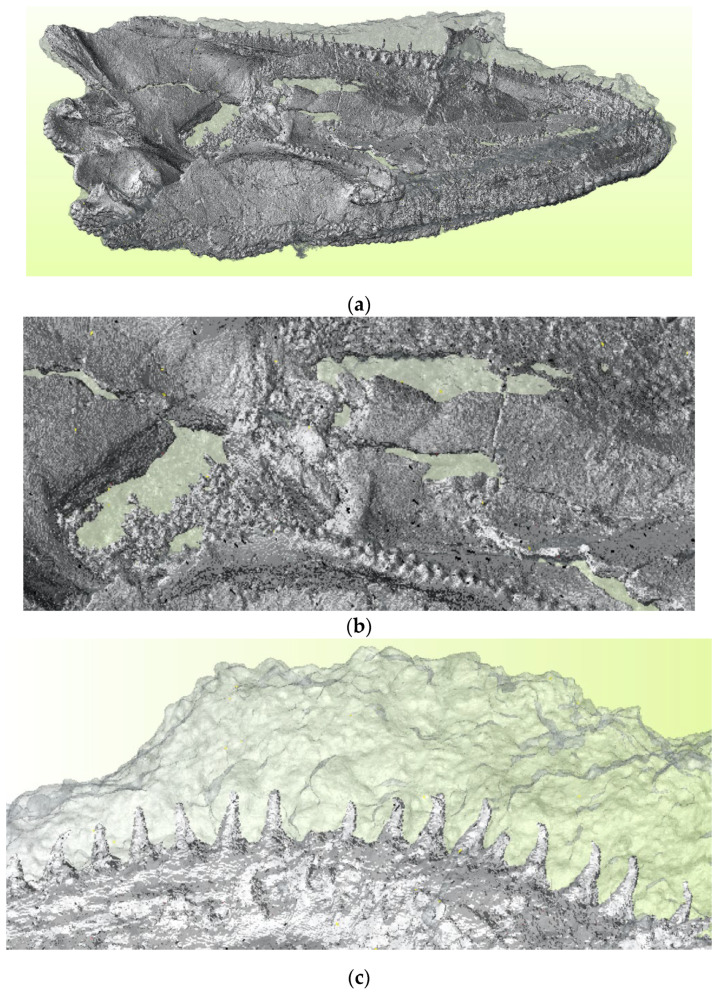
Two µCT 3D models combined together show the contrast between bone and the whole scanned object: (**a**) general appearance of the model in ventral view; (**b**) enlarged detail of the skull roof showing openings and fractures; and (**c**) fragment of the snout with teeth embedded in sediment.

The main distinctive feature of the µCT scanner compared to the optical ones is the possibility of digitally separating the fossil object from the embedding inorganic matter based on their respective density characteristics. Figure 2 presents the combination of two 3D models, one of them representing the entire volume of the object, with the other showing only the original skull without sedimentary additions. For better visualization, the whole-volume model is presented in transparent form.

In fact, proper analysis of fossil morphology can only be carried out based on a representation of the bones with the embedding sedimentary material removed digitally. Statistically, the sedimentary material had a maximal thickness of {*t_s_*}_max_ = 8.2 mm, an arithmetic mean of ${\bar{t}}_{s}$ = 0.69 mm, and a standard deviation of *σ*{*t_s_*} = 1.18 mm. Taking into account that most of the fossil is free-from embedding rock, this gives a good estimate of the sediment layer thickness in the restricted area where it is still present. This information is crucial for future attempts in mechanical or chemical preparation.

**Figure 3:**
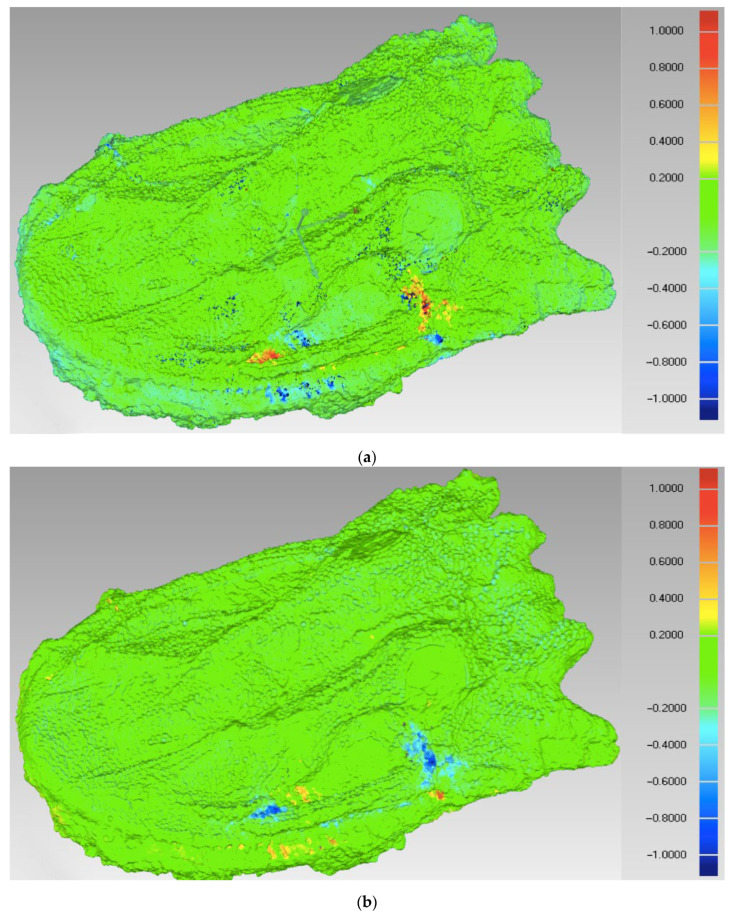
Distances between two models: (**a**) tested model YXLON against reference AICON; and (**b**) tested model AICON against reference YXLON.

However, there is no other way to assess the fidelity of the representation of fossil geometry except through its comparison with other models obtained from the scanned outer surface of the object. The models differ between one another by geometry and the number of polygons, so that statistics appear different when the reference and tested model are mutually interchanged. Thus, each pair of models had to be analyzed twice, with one of them treated as the reference and the other one as tested surface and vice versa. Figure 3 illustrates graphically the differences between AICON and YXLON models in both configurations.

The mapping shown in Figure 3 demonstrates that the vast majority of the surface exhibited distances between −0.3 and +0.3 mm, represented by the green color. Larger distances are concentrated essentially in the same places, but after reference was changed, the positive deviations (yellow and red areas) turned to negative ones (blue areas) and vice versa. However, detailed analysis revealed differences in maximal and average values, as well as in standard deviation. The values are collected in Table 4. The distances denoted ‘positive’ are placed outside the material, while the ‘negative’ ones are directed to the inside. The ‘overall average’ was calculated considering both positive and negative distances.

**Figure 4:**
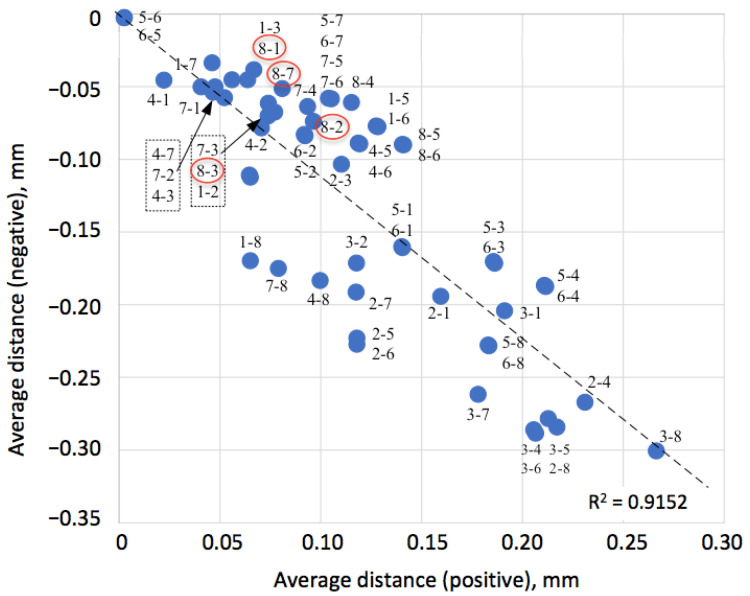
Average distances in positive (outside) and negative (inside) directions between the reference and tested models, denoted as follows: 1. AICON; 2. AR Crysta; 3. AR Strato; 4. ARTEC; 5. CREAFORM GoScan; 6. CREAFORM HandyScan; 7. EinScan Pro; and 8. YXLON.

Mainly negative values of the overall arithmetic mean indicate that the tested model surface lies predominantly inside the reference model, reflecting differences in surface details captured by different scanning technologies. Average distances of the tested model points in two directions from the reference model reflect the degree of correctness of mutual alignment of the coupled models. The diagram in Figure 4 shows the position of each couple with respect to both positive and negative distances from the reference model. Interestingly, in the area where average distances in both directions are between −0.1 mm and +0.1 mm, four couples with the YXLON model can be found (emphasized with red circles in Figure 4), but in all cases, YXLON served as a reference model. In turn, for point 3–8, where AR Strato was the reference model and YXLON was the tested one, distances in both directions reached the largest absolute values.

Visual analysis of the diagram in Figure 4 indicates a linear trend in the relations between the positive Δ*_p_* and negative Δ*_n_* distances, with the square of the correlation coefficient *R*^2^ = 0.9152. The regression model can be written as follows:

Δ*_n_* = −1.12·Δ*_p_*. (1)

Equation (1) indicates balanced mutual alignment of the models. It reflects 12% systematic offset, attributed to mesh density differences rather than scale error. The difference in the slope from 1 is insufficient and can be attributed to the differences in numbers of polygons and unsteady density of the polygons in different models. Thus, the mutual alignment of the models can be considered very successful.

It was found necessary to perform the statistical significance testing in the form of paired *t*-tests. The results are collected in Table 5. The test confirmed that all distance differences are statistically significant (*p* < 0.001). One-way ANOVA revealed a large effect, with parameters F (7.15680) = 2847.3, *p* < 0.001, and η^2^ = 0.56. Post hoc power analysis showed power > 0.95 for all comparisons (well-powered). For mean distances, 95% confidence intervals (Cis) were reported.

From the statistically significant test results, the following conclusions can be drawn:

- The pair comparison of YXLON µCT vs. AICON, with a mean distance of −0.0183 mm, approached AICON’s 20 µm specification accuracy;
- Both AR Strato comparisons showed distances below 0.03 mm, exhibiting excellent agreement. Notably, these exhibited the largest practical differences;
- CREAFORM scanners show systematic offset ca. 0.14 mm, which is still acceptable for most paleontological applications;
- All differences remained within ±0.15 mm tolerance, acceptable for museum and educational quality reproductions.

The statistical significance confirmed that the observed differences specified in Table 5 reflect genuine technological capabilities rather than measurement noise. However, practical significance must consider the application context, where differences below 0.1 mm remain suitable for most paleontological applications.

**Figure 5:**
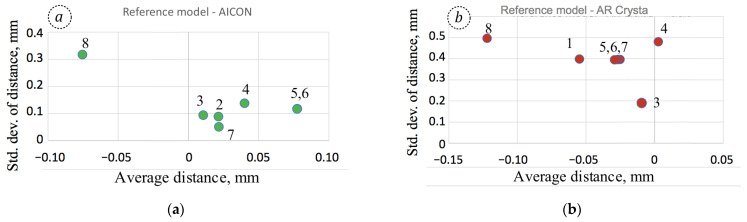

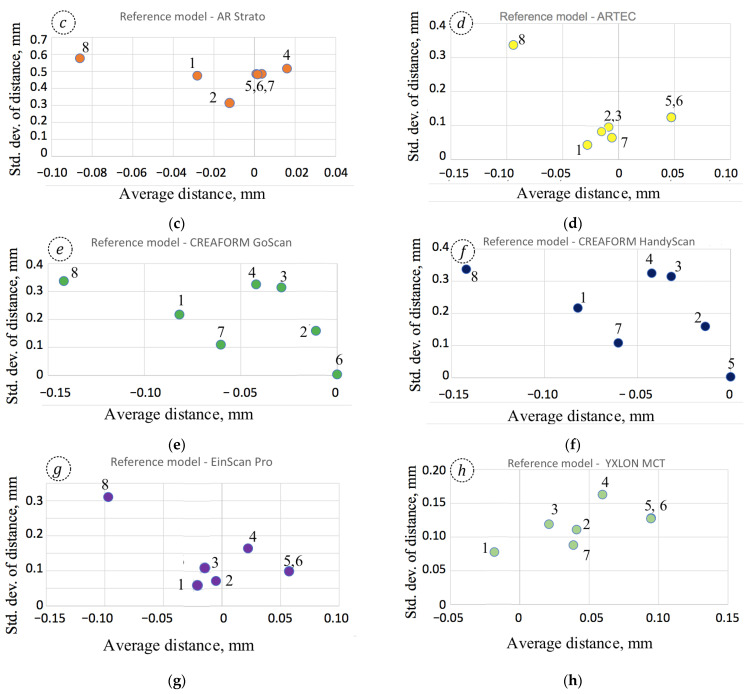
Standard deviation of distances σ{∆_d_} versus overall average distances $\bar{{∆}_{d}}$ for the pairs of models in the following order: (**a**) reference model AICON; (**b**) reference model AR Crysta; (**c**) reference model AR Strato; (**d**) reference model ARTEC; (**e**) reference model CREAFORM GoScan; (**f**) reference model CREAFORM HandyScan; (**g**) reference model EinScan Pro; and (**h**) reference model YXLON. The tested models in each diagram are denoted as follows: 1. AICON; 2. AR Crysta; 3. AR Strato; 4. ARTEC; 5. CREAFORM GoScan; 6. CREAFORM HandyScan; 7. EinScan Pro; and 8. YXLON.

For a more detailed analysis, individual diagrams were made for each reference model participating in the tests, providing the relations between average values of distances $\bar{{∆}_{d}}$ from the reference model to the tested one and the standard deviation of those distances *σ*{∆_d_}. It is worth noting that for all couples where the YXLON was tested, i.e., in Figure 5a–g, its result, denoted 8, is significantly distanced from others and is always placed in the upper left corner of the diagram.

The distanced position of the YXLON model (denoted 8 in Figure 5) from others can be attributed to the principal difference in the scanning techniques. Indirectly, this can be confirmed by large values of standard deviations *σ*{∆_d_}. In fact, the overall average distances $\bar{{∆}_{d}}$ fell between −0.075 and −0.142 mm when coupled with any of the reference models, with standard deviations between 0.31 and 0.58 mm. It is important to note that the smallest absolute values of both |$\bar{{∆}_{d}}$| = 0.0754 mm and *σ*{∆_d_} = 0.3106 mm were obtained for the reference model AICON (Figure 5a), which was ranked as the one exhibiting the highest fidelity [12]. Similarly, for the reference model YXLON (Figure 5h), AICON was the closest model, with respective values of $\bar{{∆}_{d}}$ = −0.0183 mm and *σ*{∆_d_} = 0.0778 mm. In that case, the overall average distance is consistent with the length measurement error of the AICON SmartScan system, declared in specification as 20 μm.

In general, it can be stated that the comparison of all models to the reference YXLON appeared quite positive. In particular, overall average distances were $\bar{{∆}_{d}}$ < 0.1 mm, with standard deviations of *σ*{∆_d_} < 0.2 mm. This range of inaccuracy corresponds with usual reproducibility of the typical 3D-printed copies. As was demonstrated in [23], tolerances of ±0.15 mm for the *Madygenerpeton* skull are achievable for above 75% of its scanned surface. In this respect, the YXLON µCT digital model exhibited satisfactory fidelity of the reproduction.

## 4. Practical Implications

### 4.1. General Remarks

**Figure 6:**
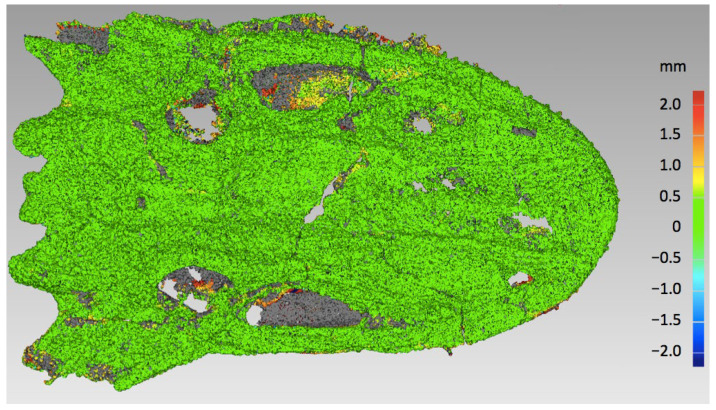
Color mapping of the sedimentary layer thickness on the fossil skull surface.

It should be noted that high-quality surface images obtained from microtomography are relatively rare and thus very valuable. For instance, the work of 12 institutions in Switzerland resulted in about 6000 images of fossil specimens, with more than 4500 of pictures made with classical photography, ca. 800 models obtained from surface scanning, and only about 450 images derived from computer tomography [24]. Apart from the high-quality surface model produced by the µCT scanner, with digitally removed sedimentary material, the present accuracy analysis provides a solid basis for further investigations on the *Madygenerpeton* skull morphology. Figure 6 presents the visualization of the sedimentary layer thickness through a comparison of two 3D models obtained from tomography, one generated from the outer surface of the object and the other from the bone surface, which is partly covered by sediment. The green color indicates medium thickness, close to zero, and it is dominating throughout the surface. The yellow and red areas indicate the thickest sediment layer on the skull surface.

Statistically, the average thickness of the sedimentary material over the *Madygenerpeton* skull was 0.69 mm, with a standard deviation of 1.18 mm. However, there are some areas of blue color, which correspond to negative thickness. These may be attributed to the inaccuracy of the models after transformation and matching, especially due to the complex surface features. Apparently, the values of differences between two digital surfaces depend on the measurement direction, which becomes critical in the case of the deformed bone surface, hidden physically under the sedimentary layer. Thus, it can be concluded that the thickness of sedimentary extraneous layers is close to zero on most of the upper surface of the object, and there are very few places where the maximal thickness of sedimentary material reaches 8.20 mm.

**Figure 7:**
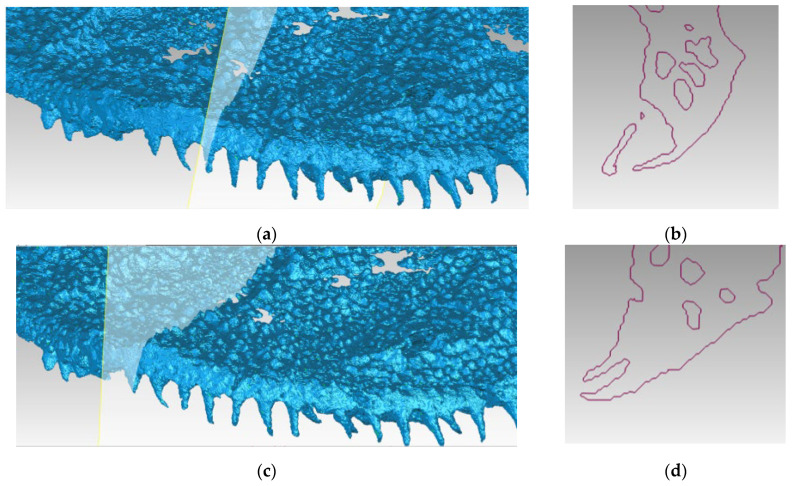
Part of the 3D surface model of the *Madygenerpeton* skull based on the YXLON µCT scan with sedimentary material removed (**a**,**c**), with the position of two virtual cross-sections showing the same bones under different angles (**b**,**d**).

The high resolution of the YXLON µCT model, with proven fidelity through comparison with optical scanners, enables detailed examination of the hidden morphological features of the fossil. Figure 7a illustrates the detailed surface area of the skull, with well distinguishable teeth, which have not been uncovered by mechanical preparation in order to avoid damaging the fossil. Visualization with different shades of blue helps to find important features and to draw relevant conclusions in paleontological terms. Moreover, virtual cross-sections can be made in arbitrary places, allowing analysis of internal structures of the fossil elements. Some exemplified sections are shown in Figure 7b,d.

The placing and curvature of the simple conical teeth that can be seen in Figure 7 allows drawing conclusions about their function and mode of replacement. This is an example of collecting and applying morphological data of vertebrates gained by µCT from hidden structures, similar to those used for phylogenetic analysis by Cunningham et al. [2] or Giles et al. [25]. Metrological analysis described in Section 2 and Section 3 proves the accuracy of those data.

**Figure 8:**
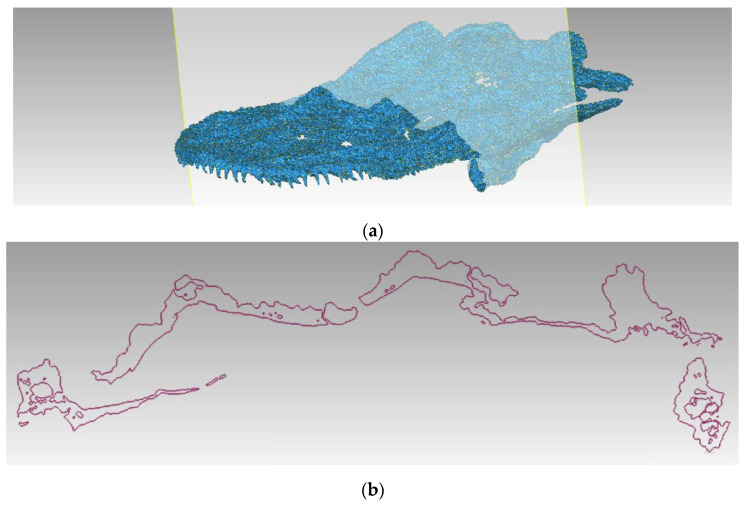
Example of the perpendicular intersection revealing damaged and fragmented skull bones of the *Madygenerpeton* fossil: (**a**) position of the intersection; and (**b**) images of the bone tissues.

In addition to the visualization of both external structures still covered by sediment and internal structures hidden within the fossil and not accessible for analysis by any other non-invasive method, µCT scanning provides models best suitable for digital restoration and retrodeformation. Fractures and deformations of the bones can be detected, as exemplified by the skull cross-section in Figure 8.

However, a detailed morphological description is beyond the objective of this research, which is focused on the accuracy of 3D digital models. Metrological analysis of the *Madygenerpeton* fossil skull ensured high fidelity of the collected data and provided a range of perspectives for further analyses. The present study reached its objective to prove the high quality of data on the morphology of the unique fossil skull collected using several scanning devices.

### 4.2. Quantitative Framework for µCT–Optical Scanner Comparison

The performed statistical analysis establishes the quantitative benchmark for µCT surface model fidelity in paleontological applications. The measured average distance of −0.0183 mm between the models obtained from YXLON µCT and AICON SmartScan is consistent with the length measurement error of the AICON SmartScan system of 20 µm, demonstrating comparable surface accuracy. Moreover, some critical thresholds were identified, as follows:

- Surface fidelity with deviations below 0.1 mm is suitable for the morphometric analysis based on digital data;
- Standard deviations below 0.2 mm are acceptable for 3D printing applications;
- In total, 75% of the surface area achieves ±0.15 mm tolerance for museum-quality reproductions.

The results also have practical significance for the industrial applications, especially in reverse engineering where freeform surfaces are to be reproduced. Especially in cases where the availability of similar scanning devices is limited, comparison can be made between the models obtained from optical scanners and the ones from tomography, with relevant fidelity analysis.

### 4.3. Digital Sediment–Fossil Separation: Quantitative Assessment

Density-based separation achieved a mean sediment thickness of 0.69 ± 1.18 mm, pointing out the areas with a maximum thickness of 8.2 mm. This quantification enables preparation of the cost–benefit analysis and choice between mechanical removal and digital separation. Based on the results, risk assessment is available for further mechanical preparation of unique specimens to avoid damage. Moreover, automated workflow can be developed for batch processing of embedded fossils.

The technical innovation of the study can be pointed out. The combination of external surface triangulation with internal density discrimination in a single scanning session eliminates the traditional choice between surface documentation and internal analysis.

### 4.4. Methodological Validation Protocol

The established statistical framework involving bilateral distance analysis with reference-tested model interchange addresses the polygon density variation problem. In particular, this study covered the YXLON model with 23.8 M polygons and the AICON model with 13.9 M polygons, and demonstrated that the analysis maintained measurement validity. The proposed protocol enables further development aimed at improving cooperation between scientific institutions, in particular

- Standardized accuracy assessment across different scanner technologies;
- Quality control for multi-institutional digitization projects;
- Validation of emerging scanning technologies against established benchmarks.

It is obvious, that the unification of the devices and scanning parameters for all paleontological objects throughout the world is impossible. With the proposed methodology, the comparative analysis between the scanned surfaces and digital models can be performed despite the differences in the available scanning devices. The results of such an analysis can be assessed from a purpose-oriented perspective, setting specific requirements for museum exhibitions, educational purposes, scientific research, etc.

The proposed methodology can be applied to various industrial tasks that involve free surface analysis and reproduction. In particular, digital twin creation, shop-floor inspection of freeform parts, or retro-deformation tasks can be solved using the proposed framework despite the polygon density variation in the available models.

Moreover, the results of the present study provide a solid basis for further analysis involving machine learning (ML) algorithms. The tasks may include sediment/bone classification, threshold prediction, etc. This research direction would require substantial collection of training data, developed across multiple fossil types and making possible algorithm validation and metrics.

### 4.5. Limitations

The presented *Madygenerpeton* skull analysis allowed for identification of some specific limitations, as follows:

- Polygon density mismatch effects were observed: YXLON µCT generated 23.8 M polygons vs. AICON’s 13.9 M, causing statistical asymmetry in bilateral comparisons. The solution will be addressed in future studies through development of polygon decimation protocols maintaining geometric fidelity while standardizing mesh density to 15 M ± 2 M polygons for all models.
- Partial surface coverage issue appeared because AR Crysta and AR Strato captured only the upper skull surface, preventing volume calculations. The solution will be addressed in future studies through implementation of minimum coverage requirements (e.g., >90% specimen surface) for inclusion in comparative studies.
- Alignment algorithm sensitivity: automatic alignment showed R^2^ ≥ 0.9 correlation, but a systematic offset in some scanner pairs. Solutions can be found by developing a hybrid alignment and combining automatic ICP with manual landmark-based registration, using certain anatomical features.
- Single specimen validation may present some limitations, since the conclusions are based on one fossil type with a specific preservation state. Possible solutions can be proposed for validation across multiple fossil categories, for example, vertebrate skulls, invertebrate shells, or plants.
- Sediment contrast dependency: digital separation succeeded due to adequate density contrast (bone vs. sediment).
- Processing workflow time disparity issues: µCT took 20 min to scan plus 4 h for reconstruction, while optical scanning took 10–15 min in total. Possible solutions may be found by implementing parallel processing pipelines, reducing reconstruction time to 1 h and less.

These targeted improvements will enable broader application of our comparative framework across paleontological collections while addressing the specific technical challenges identified in this study.

## 5. Conclusions

This study provided a novel methodology for the comprehensive statistical validation of µCT surface model fidelity for paleontological specimens, establishing quantitative accuracy benchmarks and demonstrating the feasibility of simultaneous internal–external morphological documentation. The measured accuracy parameters enabled evidence-based selection of digitization methods. The results provided a solid experimental basis for the decision-making process concerning application of several scanning methods for small paleontological objects like the fossil skull of a tetrapod. It demonstrated that morphological descriptions and reconstructions of structures hidden in sediment or within the fossil tissue could rely on the high geometrical fidelity of the YXLON µCT 3D model.

The ability of μCT to separate the 3D model of fossil bone tissue from the sedimentary material is especially important, since it is impossible using contact, optical, and laser scanning methods. The contrast in the digital model between different tissue types in μCT allows for visualization of both the external morphology and the internal structure of the fossil skull of *Madygenerpeton*.

The comparative analysis of the scanned surfaces demonstrated that the µCT model is closest to the AICON model, which was ranked as the most accurate one in a previous investigation. The overall average distance between the two models was consistent with the length measurement error of the AICON SmartScan system, declared in specification as 20 μm.

In addition, the comparison of the µCT digital surface with all tested models revealed overall average distances of $\bar{{∆}_{d}}$ < 0.1 mm with standard deviations of σ{∆_d_} < 0.2 mm. This range of inaccuracy corresponded with the usual reproducibility of typical 3D-printed copies, applicable for paleontological research and education purposes. Thus, the obtained digital models of the *Madygenerpeton* fossil skull, including that from the YXLON µCT system, exhibited satisfactory fidelity of reproduction. The data are available in the database https://doi.org/10.34740/kaggle/dsv/12365439 (accessed 1 October 2025) and can be used for further research or dissemination, either as digital models for visualization on screen or in mixed reality applications, or for additive manufacturing of replica. Moreover, further analysis of the deformed and damaged *Madygenerpeton* fossil skull may be used for the digital reconstruction of its original form.

## Figures and Tables

**Table 1:** Triangle edge length statistics.

Presence of Sediment	Min	Max	Δ	D	σ	Arithmetic Mean	Coefficient of Variation
Without sediment	9.92 × 10^−5^	1.27361	1.27351	0.001405	0.03749	0.08869	0.4227
With sediment	0.004526	1.46908	1.46455	0.00076	0.02759	0.08962	0.30786

**Table 2:** Metrological documentation of the scanners.

3D Scanner	Reference Artifact	Certified Accuracy	Resolution
AICON SmartScan-HE R5	Ball bar (150 mm, PTB certified)	20 µm + 6 µm/m	0.05 mm
Mitutoyo AR Crysta Plus M574	Gauge block set (Grade 0, NPL)	(2+L/300) µm	0.02 mm
Mitutoyo AR Strato Apex 544	Ring gauge (Ø50 mm, NIST)	(1.5+L/350) µm	0.015 mm
ARTEC Space Spider	Geometric calibration target	0.1 mm	0.2 mm
CREAFORM HandyScan 3D 700	VDI/VDE 2634 test object	0.030 mm	0.05 mm
CREAFORM GoScan 3D 20	Step height standard	0.050 mm	0.1 mm
EinScan Pro 2X Plus	Manufacturer sphere bar	0.05 mm	0.2 mm

**Table 3:** Basic characteristics of the digital models created on the data obtained from different scanners.

3D Scanner	Model Characteristics			
	Number of Polygons, pcs	Dimensions Along the Coordinate Axes *X*, *Y*, and *Z*, mm	Surface Area, mm^2^	Volume of the Model, mm^3^
AICON [12]	13,913,354	109.2 × 31.6 × 68.8	19,307.587	30,571.640
Mitutoyo AR Strato [12]	230,378	106.3 × 20.1 × 65.1	8180.969	–
YXLON µCT	23,868,690	108.9 × 31.6 × 68.7	21,562.233	29,662.658

**Table 4:** Statistics of the distances between the 3D models.

Reference Model	Test Model	Distance Statistics, mm					
		Maximum		Arithmetic Mean			Standard Deviation
		Positive	Negative	Overall	Positive	Negative	
AICON	YXLON µCT	1.9986	−1.9999	−0.0754	0.0652	−0.1698	0.3172
YXLON µCT	AICON	0.9459	−1.2649	−0.0183	0.0638	−0.0454	0.0778
AR Crysta	YXLON µCT	2.0000	−2.0000	−0.1221	0.2129	−0.2784	0.4966
YXLON µCT	AR Crysta	1.5566	−1.9985	0.0409	0.0964	−0.0740	0.1110
AR Strato	YXLON µCT	2.000	−2.000	−0.0860	0.2664	−0.3006	0.5762
YXLON µCT	AR Strato	1.9899	−1.9981	0.0210	0.0772	−0.0676	0.1191
ARTEC	YXLON µCT	2.0000	−1.9999	−0.0941	0.0998	−0.1835	0.3369
YXLON µCT	ARTEC	1.9998	−1.9999	0.0596	0.1153	−0.0609	0.1630
CREAFORM GoScan	YXLON µCT	1.9996	−2.000	−0.1418	0.1834	−0.2284	0.3374
YXLON µCT	CREAFORM GoScan	1.0322	−0.9410	0.0946	0.1412	−0.0901	0.1281
CREAFORM HandyScan	YXLON µCT	1.9986	−2.0000	−0.1421	0.1831	−0.2278	0.3370
YXLON µCT	CREAFORM HandyScan	1.0296	−0.9519	0.0945	0.1405	−0.0899	0.1274
EinScan Pro	YXLON µCT	1.9959	−2.0000	−0.0970	0.0791	−0.1751	0.3106
YXLON µCT	EinScan Pro	0.9548	−1.0920	0.0386	0.0810	−0.0514	0.0881

**Table 5:** Statistical significance testing results.

Scanner Pair Comparison	Mean Distance, mm	*p*-Value (Paired *t*-Test)	95% CI	Effect Size (Cohen’s *d*)	Practical Significance
AICON vs. YXLON µCT	−0.0754	*p* < 0.001	[−0.082, −0.069]	d = 0.24 (small)	Significant *
YXLON µCT vs. AICON	−0.0183	*p* < 0.001	[−0.024, −0.013]	d = 0.23 (small)	Practically equivalent
AR Crysta vs. YXLON µCT	−0.1221	*p* < 0.001	[−0.135, −0.109]	d = 0.25 (small)	Significant *
YXLON µCT vs. AR Crysta	0.0409	*p* < 0.001	[0.031, 0.051]	d = 0.37 (small)	Significant *
AR Strato vs. YXLON µCT	−0.0860	*p* < 0.001	[−0.105, −0.067]	d = 0.15 (small)	Significant *
YXLON µCT vs. AR Strato	0.0210	*p* < 0.001	[0.011, 0.031]	d = 0.18 (small)	Significant *
ARTEC vs. YXLON µCT	−0.0941	*p* < 0.001	[−0.107, −0.081]	d = 0.28 (small)	Significant *
YXLON µCT vs. ARTEC	0.0596	*p* < 0.001	[0.046, 0.073]	d = 0.37 (small)	Significant *
CREAFORM GoScan vs. YXLON µCT	−0.1418	*p* < 0.001	[−0.155, −0.128]	d = 0.42 (medium)	Highly Significant **
YXLON µCT vs. CREAFORM GoScan	0.0946	*p* < 0.001	[0.081, 0.108]	d = 0.74 (medium)	Highly Significant **
CREAFORM HandyScan vs. YXLON µCT	−0.1421	*p* < 0.001	[−0.156, −0.128]	d = 0.42 ( medium)	Highly Significant **
YXLON µCT vs. CREAFORM HandyScan	0.0945	*p* < 0.001	[0.080, 0.109]	d = 0.74 (medium)	Highly Significant **
EinScan Pro vs. YXLON µCT	−0.0970	*p* < 0.001	[−0.110, −0.084]	d = 0.31 (small)	Significant *
YXLON µCT vs. EinScan Pro	0.0386	*p* < 0.001	[0.028, 0.049]	d = 0.44 (medium)	Significant *

## Data Availability

The data are available in the database https://doi.org/10.34740/kaggle/dsv/12365439 (accessed 1 October 2025).
